# Multicenter analysis of epidemiological and clinical features of pediatric acute lower respiratory tract infections associated with common human coronaviruses in China, 2014–2019

**DOI:** 10.1186/s12985-023-02198-6

**Published:** 2023-10-10

**Authors:** Shuaibing Han, Baoping Xu, Qianyu Feng, Ziheng Feng, Yun Zhu, Junhong Ai, Li Deng, Changchong Li, Ling Cao, Yun Sun, Zhou Fu, Rong Jin, Yunxiao Shang, Zhiming Chen, Lili Xu, Zhengde Xie, Kunling Shen

**Affiliations:** 1 Beijing Key Laboratory of Pediatric Respiratory Infection Diseases, Key Laboratory of Major Diseases in Children, Ministry of Education, National Clinical Research Center for Respiratory Diseases, National Key Discipline of Pediatrics (Capital Medical University), Beijing Pediatric Research Institute, Beijing Children’s Hospital, Capital Medical University, National Center for Children’s Health, Beijing, 100045 China; 2 Research Unit of Critical Infection in Children, Chinese Academy of Medical Sciences, 2019RU016, Beijing, 100045 China; 3 Department of Respiratory Diseases I, Beijing Children’s Hospital, Capital Medical University, National Clinical Research Center for Respiratory Diseases, National Center for Children’s Health, Beijing, 100045 China; 4 Guangzhou Women and Children’s Medical Center, Guangzhou, 510623 China; 5 The 2nd Affiliated Hospital and Yuying Children’s Hospital of Wenzhou Medical University, Wenzhou, 325027 China; 6 Children’s Hospital, Capital Institute of Pediatrics, Beijing, 100020 China; 7 Yinchuan Maternal and Child Health Hospital, Yinchuan, 750000 China; 8 Children’s Hospital of Chongqing Medical University, Chongqing, 400015 China; 9 Guiyang Women and Children Healthcare Hospital, Guiyang, 550003 China; 10 Shengjing Hospital of China Medical University, Shenyang, 110004 China; 11 The Children’s Hospital of Zhejiang University School of Medicine, Hangzhou, 310005 China

**Keywords:** Common human coronavirus, Children, Epidemiology, Clinical manifestation, Multicentre research

## Abstract

The common human coronaviruses (HCoVs) HCoV-229E, HCoV-OC43, HCoV-NL63, and HCoV-HKU1 which are members of the coronavirus family are long co-existed with humans and widely distributed globally. Common HCoVs usually cause mild, self-limited upper respiratory tract infections (URTI), and also associated with lower respiratory tract infections (LRTI), especially in children. However, there are little multicentre studies have been conducted in children of several different areas in China, and the epidemic potential of common HCoVs remains unclear. Understanding of the common HCoVs is valuable for clinical and public health. Herein, we retrospectively analysed the medical records of children with acute lower respiratory tract infection admitted to 9 hospitals from different regions in China from 2014 to 2019. Of the 124 patients who tested positive for coronaviruses, OC43 was the predominant type, accounting for 36.3% (45/124) of the detections. Children aged ≤ 6 months and 12–23 months had the highest detection rate of common HCoVs, and the detection rate gradually declined after 2 years old. These four HCoVs could be detected all year round. Among the areas of our study, the overall positive rate was higher in southern China, especially in Guangzhou (29/124, 23.4%). Moreover, common HCoV-positive patients were codetected with 9 other common respiratory pathogens. 229E (11/13, 84.6%) was the most frequently associated with codetection, with EV/RhV was the most frequently codetected virus. Cough (113/124, 91.1%) and fever (73/124, 58.9%) were the most common symptoms of common HCoVs infection.

## Introduction

Human coronaviruses (HCoVs), which are enveloped, positive-strand RNA viruses belonging to the Coronaviridae family [1–3], are the main causes of respiratory tract infections ranging from subclinical and mild infections (such as the common cold) to severe acute respiratory syndrome (such as pneumonia and bronchitis) [3–8]. The first human coronavirus was detected in the mid-1960s [9, 10]. To date, seven HCoVs have been detected: human coronavirus 229E (HCoV-229E, 1962), human coronavirus OC43 (HCoV-OC43, 1967), severe acute respiratory syndrome coronavirus (SARS-CoV, 2002), human coronavirus NL63 (HCoV-NL63, 2004), human coronavirus HKU1 (HCoV-HKU1, 2005), middle east respiratory syndrome coronavirus (MERS-CoV, 2012), and severe acute respiratory syndrome coronavirus 2 (SARS-CoV-2, 2019) [3, 11–13]. Coronaviruses (CoVs) are separated into four genera bases on its genomic structures and phylogenetic relationships: alpha-, beta-, gamma- and delta-coronaviruses [1]. Of these, NL63 and 229E belong to the genus alpha-coronavirus, while OC43 and HKU1 are members of the genus beta-coronavirus, which also includes SARS-CoV, MERS-CoV and SARS-CoV-2 [5, 6, 11–13].

The common HCoVs also called “seasonal HCoVs” which include 229E, OC43, NL63 and HKU1 have long co-existed with humans, especially in children [5, 14]. As mentioned earlier, there are many similarities between SARS-CoV-2 and common HCoVs infections in children. This four HCoVs species are distributed globally and with seasonal patterns [2, 3], meanwhile, the prevalence of them is varies by region, country and time [4, 14–21]. In general, OC43 and NL63 are the most commonly HCoV, while HKU1 and 229E are relatively less [22, 23]. They are more prevalent during the spring and winter months [3, 14, 16, 21], while a study of NL63 infection in Hong Kong showed an epidemic trend from spring to summer [19]. The common HCoVs were low pathogenic and usually cause mild-moderate respiratory tract diseases [5, 13]. It is one of the most frequently detected aetiologic agent of the seasonal common cold [24], and most frequently causes a wide range of upper respiratory tract infections (URTIs), characterised by cough, nasal congestion, and rhinorrhoea, as well as mild fever [16, 18, 25]. It also results in lower respiratory tract illness (LRTI), including bronchiolitis and pneumonia, eventually leading to hospitalisation, especially in immunocompromised children and infants [2, 3, 16, 18, 25–28]. Several studies have indicated that OC43 and 229E are associated with mild, self-limiting upper respiratory tract diseases, while NL63 and HKU1 are associated with lower respiratory tract diseases and more serious respiratory tract infections [2, 3, 17]. In addition, approximately 30–50% of common HCoV infections are codected with other respiratory pathogens [14, 29].

Until now, although some research has analysed the clinical or epidemiological characteristics of common HCoV infections, these studies just limit to a certain area in China. However, multicentre studies focusing on children from different regions in China at the same time are rare [14, 18, 19]. The epidemic potential of common HCoVs remains unclear. Hence, this multicentre study aimed at characterising the epidemiological, clinical and codetection characteristics of common HCoV infections in children admitted to hospitals of various regions in China. The results of this study will assist in the development of prevention and control strategies for common HCoV infections in children.

## Materials and methods

### Patient enrolment

We performed a multicentre retrospective study of hospitalised children under the age of 18 years with acute lower respiratory tract infections admitted to various hospitals in China from January 2014 to December 2019. Chest X-rays were taken for all children. The diagnosis of pneumonia relies on the identification of a novel infiltrate on chest X-rays, accompanied by at least one of the subsequent symptoms: hypothermia (temperature < 35.0 °C) or fever (temperature ≥ 38.0 °C), pleuritic chest pain, the onset of a new cough with or without sputum production, or discernible changes in breath sounds during auscultation. While the diagnosis of bronchopneumonia based on acute onset and the presence of respiratory symptoms and signs, as well as chest X-rays revealed irregular patchy shadows distributed along the lung texture, with light and fuzzy edge density, and no signs of consolidation [30]. Patients were enrolled from 9 hospitals in 8 cities in 6 different areas of China: Beijing Children's Hospital in Beijing (central China), Capital Institute of Paediatrics in Beijing (central China), Yinchuan Maternal and Child Health Hospital in Yinchuan (northwestern China), Shengjing Hospital of China Medical University in Shenyang (northeastern China), Guangzhou Women and Children’s Medical Center in Guangzhou (southern China), Children's Hospital of Chongqing Medicine University in Chongqing (southwestern China), Guiyang Women and Children Health care Hospital in Guiyang (southwestern China), Yuying Children’s Hospital of Wenzhou Medical University in Wenzhou (southeastern China), and The Children's Hospital of Zhejiang University School of Medicine in Hangzhou (southeastern China). Patients with nosocomial respiratory infections, inhaled airway foreign bodies, and acute upper respiratory infections were excluded.

### Specimens and clinical information collection

Enrolled children and/or their caregivers were interviewed using a standardised questionnaire, and medical charts were abstracted after discharge. Demographic, epidemiologic, and clinical data were systematically collected. Patient clinical records and information were anonymised and de-identified prior to analysis. Nasopharyngeal aspirates (NPAs) specimens were collected using sterile flocked Dacron swabs with flexible shafts. Swabs were passed through one nostril to the nasopharynx and rotated to collect epithelial tissue and absorb secretions. Swabs were combined in 3 mL sterile universal transport medium. All specimens were obtained in the first 24 h of hospitalisation and stored frozen (− 80 °C) until analysis. All patients were enrolled in a network that monitored viral pathogens of children with acute lower respiratory tract infections, and all the patients’ information was submitted to the Infectious Disease Surveillance System of China. This study was performed in strict accordance with the human subject protection guidance and was approved by the Ethical Review Committees of Beijing Children’s Hospital, Capital Institute of Pediatrics, Yinchuan Maternal and Child Health Hospital, Shengjing Hospital of China Medical University, Guangzhou Women and Children’s Medical Center, Children's Hospital of Chongqing Medicine University, Guiyang Women and Children Healthcare Hospital, Yuying Children’s Hospital of Wenzhou Medical University, and The Children's Hospital of Zhejiang University School of Medicine. Written informed consent was obtained from the participants (≥ 8 years) or their parents/guardian (< 8 years).

### Molecular detection

Viral nucleic acids in the clinical samples were extracted using the NucliSens easyMAG system (bioMérieux, Marcy-l’Etoile, France) according to the manufacturer’s instructions. The Luminex xTAG respiratory viral panel (RVP) assay and Luminex 200 instrument (Luminex, Austin, TX) were used to detect 18 common respiratory viral pathogens and subtypes in the nucleic acid samples, including HMPV, influenza A, influenza A subtype H1, influenza A subtype H3, 2009 H1N1, influenza B, human adenoviruses (HAdVs), human parainfluenza virus (HPIV) 1–4, respiratory syncytial virus (RSV) A and B, enteroviruses and rhinoviruses (EVs/RhVs), human coronavirus (HCoV) HKU1, 229E, NL63 and OC43, and human bocavirus (HBoV). An internal positive control (MS2) was added to each specimen before nucleic acid extraction, and a positive PCR control (Lambda DNA) was added in every PCR batch run, following the manufacturer’s manual. Bacterial infection tests were not performed in our study.

### Statistical analysis

Statistical analyses were performed using SPSS version 19.0 (SPSS Inc., Chicago, IL). The continuous variables between two groups were compared using a two-tailed independent-samples t test. The differences in the detection rate of HCoVs in children of different sexes, age groups, years and areas were compared using χ^2^ tests or Fisher’s exact test. A value of *P* < 0.05 was considered statistically significant.

## Results

### Detection of HCoVs among enrolled patients

**Fig. 1:**
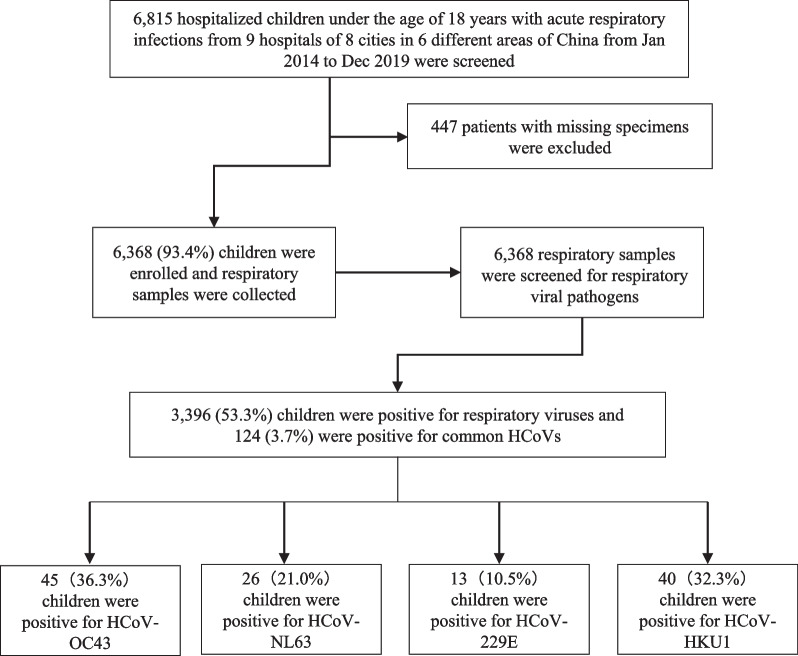
Flowchart of enrolled children and the detection of respiratory virus

Of the 6815 hospitalised children’s nasopharyngeal swab samples, we excluded 447 patients with missing specimens, and 6368 (93.4%) patients were finally enrolled in this study. A flowchart of our enrolled patients is shown in Fig. 1. Respiratory viruses were detected in 3396 of the 6368 (53.3%) children, and common HCoVs were detected in 124 of the 3396 (3.7%) children with respiratory virus infection. Among all the common HCoV-infected patients, 45 (36.3%) were positive for OC43, 26 (21.0%) for NL63, 13 (10.5%) for 229E and 40 (32.3%) for HKU1. Overall, our results showed that the most common HCoV was OC43, followed by HKU1, NL63 and 229E.

### Sex and age distribution characteristics of HCoV-positive patients

Among all HCoV-infected children, 93 (75.0%) were male, and the ratio of males to females was 3:1 (93:31). There were no significant differences in children’s sex between these four HCoVs (*p* = 0.939).

**Fig. 2:**
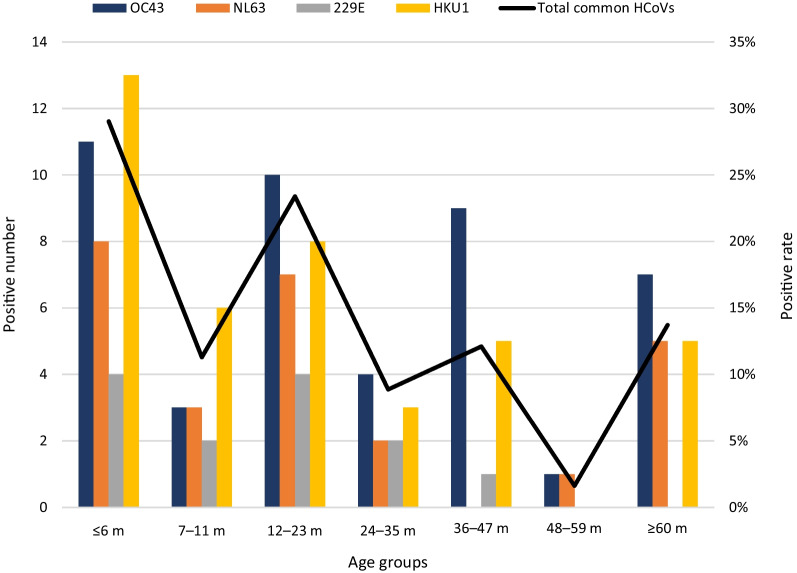
Age distribution of children with human coronaviruses OC43, NL63, 229E and HKU1. The left axis showed the total number of positive cases and the right axis showed the positive rate of total common HCoVs. Total common HCoVs: total positive rate of OC43, NL63, 229E and HKU1. M: month(s)

The median age of the common HCoV-positive cases was 1.25 years (interquartile range [IQR]: 0.5–3 years), and the patients who were positive for a single HCoV species were as follows: OC43, 1.75 years (interquartile range [IQR]: 0.56–3.19 years); NL63, 1.08 years (interquartile range [IQR]: 0.5–2.25 years); 229E, 0.92 years (interquartile range [IQR]: 0.08–1.83 years); and HKU1, 1 year (interquartile range [IQR]: 0.5–2.87 years). Patients with common HCoV positivity aged ≤ 6 months and 12–23 months had the highest detection rate, which began to decline after 24 months (Fig. 2). Similarly, for OC43, NL63, 229E, and HKU1, the highest positive rates were also in the ≤ 6 months and 12–23 months age groups (Fig. 2). No significant differences were observed between these age groups (*P* = 0.958) (Table 1).

**Table 1:** Clinical characteristics of common human coronavirus-positive patients

Parameter	No. events detected in 124 children tested (%)					
	OC43-Positive (%, n = 45)	NL63-Positive (%, n = 26)	229E-Positive (%, n = 13)	HKU1-Positive (%, n = 40)	Common-HCoVs-Positive (%, n = 124)	*P* Value
Age-year						
Median	1.75	1.08	0.92	1	1.25	
Interquartile range	0.56–3.19	0.5–2.25	0.08–1.83	0.5–2.87	0.5-3	
Age group-no. (%)						0.958
≤ 6 m	11 (24.4)	8 (30.8)	4 (30.8)	13 (32.5)	36 (29.0)	0.062
7–11 m	3 (6.7)	3 (11.5)	2 (15.4)	6 (15.0)	14 (11.3)	0.064
12–23 m	10 (22.2)	7 (26.9)	4 (30.8)	8 (20.0)	29 (23.4)	0.060
24–35 m	4 (8.9)	2 (7.7)	2 (15.4)	3 (7.5)	11 (8.9)	0.060
36–47 m	9 (20.0)	–	1 (7.7)	5 (12.5)	15 (12.1)	0.095
48–59 m	1 (2.2)	1 (3.8)	–	–	2 (1.6)	0.099
≥ 60 m	7 (15.6)	5 (19.2)	–	5 (12.5)	17 (13.7)	0.072
Sex- no. (%)						0.939
Male	35 (77.8)	20 (76.9)	10 (76.9)	28 (70.0)	93 (75.0)	0.063
Female	10 (22.2)	6 (23.1)	3 (23.1)	12 (30.0)	31 (25.0)	0.064
Clinical symptoms-no. (%)						0.045
Cough	38 (84.4)	22 (84.6)	13 (100.0)	40 (100.0)	113 (91.1)	0.063
Fever	31 (68.9)	17 (65.4)	5 (38.5)	20 (50.0)	73 (58.9)	0.067
Expectoration	7 (15.6)	3 (11.5)	–	–	10 (8.1)	0.113
Wheeze	12 (26.7)	–	4 (30.8)	3 (7.5)	19 (15.3)	0.094
Haemoptysis	–	3 (11.5)	–	2 (5.0)	5 (4.0)	0.103
Rash	–	–	–	2 (5.0)	2 (1.6)	0.178
Admitting diagnosis-no. (%)						0.296
Pneumonia	16 (35.6)	15 (57.7)	5 (38.5)	19 (47.5)	55 (44.4)	0.062
Bronchopneumonia	21 (46.7)	6 (23.1)	6 (46.2)	15 (37.5)	48 (38.7)	0.068
Mycoplasma pneumonia	–	3 (11.5)	–	1 (2.5)	4 (3.2)	0.120
Bronchitis	3 (6.7)	–	–	2 (5.0)	5 (4.0)	0.103
Bronchiolitis	1 (2.2)	2 (7.7)	1 (7.7)	2 (5.0)	6 (4.8)	0.061
Viral pneumonia	–	–	1 (7.7)	1 (2.5)	2 (1.7)	0.099
Uncertainty	4 (8.9)	–	–	–	4 (3.2)	0.391

### Regional distribution characteristics of HCoV-positive patients

**Fig. 3:**
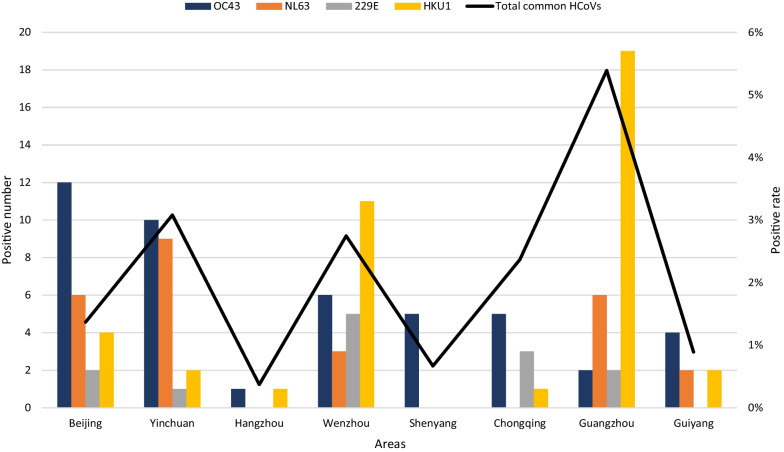
Regional distribution of children with human coronaviruses OC43, NL63, 229E and HKU1. The left axis showed the positive number of each virus and the right axis showed the local positive rate of total common HCoVs. Total common HCoVs: total positive rate of OC43, NL63, 229E and HKU1

**Fig. 4:**
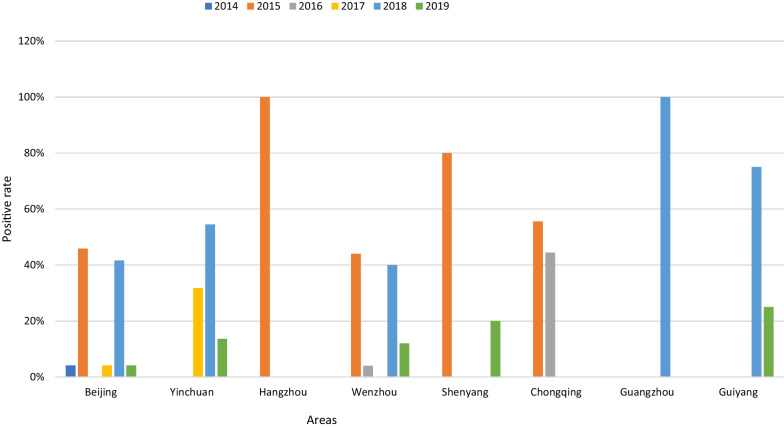
Regional distribution of human coronaviruses OC43, NL63, 229E and HKU1 in patients from January 2014 to December 2019

**Fig. 5:**
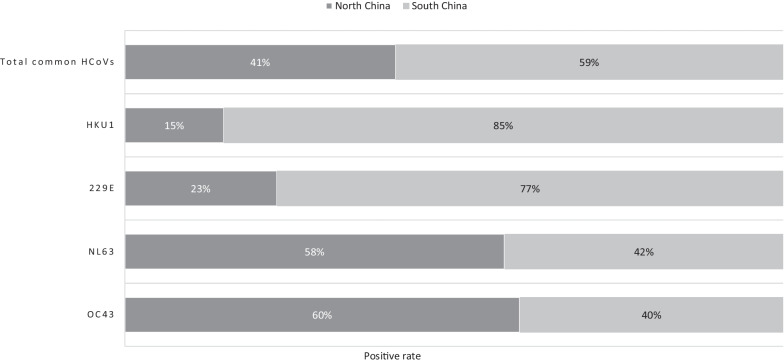
Detection rate of the four HcoVs in children in northern and southern China. Total common HcoVs: total positive rate of OC43, NL63, 229E and HKU1. HcoVs: human coronaviruses. Northern China included Beijing, Shenyang and Yinchuan. Southern China included Wenzhou, Hangzhou, Chongqing, Guangzhou, and Guiyang

The detection rate of HCoV varied in different regions (*P* = 0.019, Fig. 3). According to the difference in regions, statistically significant differences were observed in the detection rate of common HCoVs (*P* = 0.042), OC43 (*P* = 0.004), NL63 (*P* = 0.039) and 229E (*P* = 0.035). Regarding common HCoVs, Guangzhou showed the highest detection rate, with a positive rate up to 5.39% (29/538), followed by Yinchuan at 3.08% (22/714), Wenzhou at 2.75% (25/910), Chongqing at 2.37% (9/380), Beijing at 1.37% (24/1753), Guiyang at 0.89% (8/897), Shenyang at 0.67% (5/748), and Hangzhou at 0.37% (2/538). The region with the highest positive rate of OC43 was Beijing (26.7% (12/45)), followed by Yinchuan (22.2% (10/45)). The highest positive rate of NL63 was in Yinchuan (34.6% (9/26)), followed by Beijing (23.1% (6/26)) and Guangzhou (23.1% (6/26)). The highest positive rate of 229E was in Wenzhou (38.5% (5/13)), followed by Chongqing (23.1% (3/13)). The highest positive rate of HKU1 was in Guangzhou (47.5% (19/40)), followed by Wenzhou (27.5% (11/40)) (Fig. 3). Furthermore, the detection rates of the common HCoVs in Beijing and Wenzhou in 2015 and 2018 were both higher, while those in Shenyang, Hangzhou and Chongqing were higher in 2015, and those in Guangzhou, Yinchuan, Guiyang were higher in 2018 (Fig. 4). The detection rate of common HCoV infection seems to be higher in southern China (58.9%, 73/124) than in northern China (41.1%, 51/124), as deduced from our results (Fig. 5). There were significant differences in the detection rate of HCoV between northern and southern China (*P* = 0.0002). For OC43 (60.0%, 27/45) and NL63 (57.7%, 15/26), the positive rate was higher in northern China, but 229E (76.9%, 10/13) and HKU1 (85.0%, 34/40) were higher in southern China (Fig. 5).

### Seasonal distribution characteristics of HCoV-positive cases

**Fig. 6:**
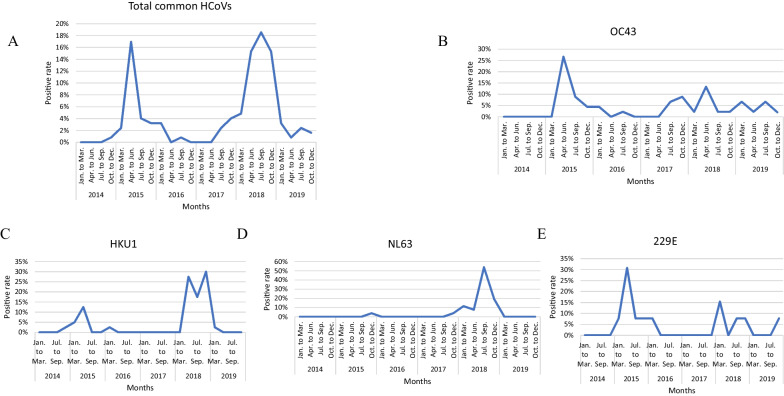
Monthly distribution of human coronaviruses OC43, NL63, 229E and HKU1 in patients between January 2014 and December 2019. **A** Monthly distribution of total positive rate of OC43, NL63, 229E and HKU1. HCoVs: human coronaviruses. **B** Monthly distribution of OC43. **C** Monthly distribution of HKU1. **D** Monthly distribution of NL63. **E** Monthly distribution of 229E

**Fig. 7:**
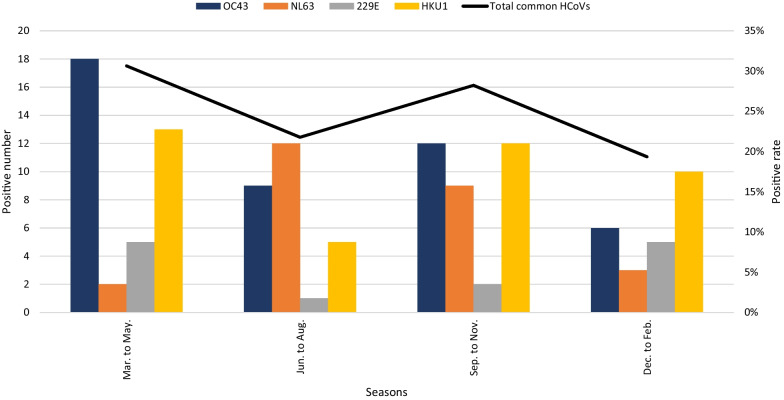
Seasonal distribution of human coronaviruses OC43, NL63, 229E and HKU1 in patients from January 2014 to December 2019. The year was divided into spring (March–May), summer (June–August), autumn (September–November), and winter (December–February)

The positive rate of the common HCoVs varied throughout each year (*P* < 0.001), was highest in 2018 (54.0%, 67/124) and lowest in 2014 (0.8%, 1/124) and demonstrated a distinct peak in 2018 and a slightly lower peak in 2015 (Fig. 6A). OC43 was mainly detected in 2015 (40.0%, 18/45) (*P* = 0.037), 229E was mainly detected in 2015 (53.8%, 7/13), and NL63 (92.3%, 24/26) and HKU1 (75.0%, 30/40) were mainly detected in 2018 (Fig. 6B–E). OC43, NL63, 229E, and HKU1 all peaked in 2015 and 2018, although the peaks varied. Furthermore, the positive rates of OC43 and 229E fluctuated from 2015 to 2019. NL63 demonstrated a distinct peak and a small peak in 2018, with a less pronounced peak in 2015. HKU1 demonstrated two peaks in 2018 and a small peak in 2015, with a less pronounced peak in 2014. To learn more about the seasonality of the four HCoVs, the year was divided into March–May (spring), June–August (summer), September–November (autumn), and December-February (winter). There were significant differences in the detection rate of each HCoV (*P* = 0.008, Fig. 7), common HCoVs (*P* = 0.003), OC43 (*P* = 0.022) and HKU1 (*P* = 0.011) between various seasons. Our results illustrate that OC43 and 229E were mainly detected in spring, with some cases detected in autumn and winter; NL63 was mainly detected in summer and autumn and some in winter. The positive rate of HKU1 was high in every season except for summer, which showed a slightly lower rate.

### Codetection with other respiratory viruses

Children who were HCoV-positive were also tested for 9 other common respiratory pathogens: enterovirus/rhinovirus, human bocavirus, herpes simplex virus, respiratory syncytial virus, human metapneumovirus, mycoplasma pneumoniae, human adenovirus, parainfluenza viruses and influenza viruses (Table 2). There were no statistically significant differences observed in the detection rate of HCoV when comparing single detected and codetected cases (*P* = 0.558) (Table 2). Out of the 124 HCoV-positive patients, 32.3% (40/124) were detected with only one HCoV strain, whereas 67.7% (84/124) were identified as being codetected with one or more other strains of HCoV or another respiratory pathogen (Table 2). The highest rate of codetection was 229E (84.6%, 11/13), followed by HKU1 (70.0%, 28/40), OC43 (66.7%, 30/45) and NL63 (57.7%, 15/26). Among them, one non-HCoV respiratory virus was codetected in 64 (76.2%, 64/84) patients, and two and three non-HCoV respiratory viruses were codetected in 16 (19.0%, 16/84) and 4 (4.8%, 4/84) patients, respectively. For OC43, NL63, 229E, and HKU1, the rates of dual infections were 80.0% (24/30), 80.0% (12/15), 72.7% (8/11), 71.4% (20/28), respectively. Moreover, EV/RhV was the most common codetected pathogen, followed by HBoV (Table 2). In addition to the most frequent codetected virus of HKU1 being HBoV, the most frequent codetected virus of OC43, NL63 and 229E was EV/RhV. The codetection rate of EV/RhV and HBoV was higher with OC43 and HKU1, respectively, than with other types of HCoV. Surprisingly, only OC43 was codetected with other HCoVs (two children codetected with OC43 and 229E and one child codetected with OC43 and HKU1), no NL63 and other HCoV codetected were detected, and HAdV was only codetected with HKU1.

**Table 2:** Codetection of common HCoVs and other respiratory viruses in the study

	OC43		NL63		229E		HKU1		Common HCoVs
	Codetection viruses	No (%, n = 45)	Codetection viruses	No (%, n = 26)	Codetection viruses	No (%, n = 13)	Codetection viruses	No (%, n = 40)	No (%, n = 124)
Single detection		15 (33.3)		11 (42.3)		2 (15.4)		12 (30)	40 (32.3)
Codetection									
1 Virus									
	EV/RhV	10 (22.2)	EV/RhV	4 (15.4)	EV/RhV	3 (23.1)	HBoV	9 (22.5)	64 (51.6)
	HMPV	4 (8.9)	HBoV	3 (11.5)	RSV	3 (23.1)	EV/RhV	5 (12.5)	
	PIV	3 (6.7)	RSV	1 (3.8)	OC43	1 (7.7)	PIV	2 (5.0)	
	HBoV	2 (4.4)	HMPV	1 (3.8)	Flu	1 (7.7)	RSV	2 (5.0)	
	RSV	2 (4.4)	Flu	1 (3.8)			OC43	1 (2.5)	
	HKU1	1 (2.2)	PIV	1 (3.8)			MP	1 (2.5)	
	229E	1 (2.2)	HRV	1 (3.8)					
	HRV	1 (2.2)							
2 Viruses									
	EV/RhV + 229E	1 (2.2)	EV/RhV + MP	3 (11.5)	EV/RhV + PIV	1 (7.7)	HAdV + HBoV	4 (10)	16 (12.9)
	EV/RhV + HMPV	1 (2.2)			EV/RhV + OC43	1 (7.7)	PIV + HMPV	1 (2.5)	
	Flu + MP	1 (2.2)					PIV + HAdV	1 (2.5)	
	EV/RhV + PIV	1 (2.2)							
	HRV + PIV	1 (2.2)							
3 Viruses									
	RSV + HMPV + HRV	1 (2.2)			RSV + EV/RhV + HBoV	1 (7.7)	HBoV + Flu + HAdV	1 (2.5)	4 (3.2)
							RSV + Flu + HAdV	1 (2.5)	
Total		45		26		13		40	124

### Clinical presentations of HCoV-positive patients

HCoV infections are accompanied by a variety of clinical manifestations. Most patients presented with mild clinical symptoms, with cough and fever being the most common symptoms of HCoV infection (Table 3). There were significant differences in children’s clinical presentations (*P* = 0.045). Of these, patients with 229E and HKU1 infection all developed symptoms of coughing, and only HKU1 infection was accompanied by rash. HCoV infection was mainly associated with various pneumonias. Patients with common HCoV positivity were diagnosed with pneumonia (44.4%), bronchopneumonia (38.7%), mycoplasma pneumonia (3.2%), bronchitis (4.0%), bronchiolitis (4.8%), viral pneumonia (1.7%), and some uncertainty (3.2%). The most frequent diagnosis was pneumonia, followed by bronchopneumonia. Compared with other HCoV, the most common diagnosis of OC43 and 229E infection was bronchopneumonia (46.7% and 46.2%, respectively), while the most common diagnosis of NL63 and HKU1 infection was pneumonia (57.7% and 47.5%, respectively). Furthermore, comparing single HCoV detected and HCoV condetected cases, the most common clinical presentations was cough (85.0% and 94.0%, respectively), and the most common clinical diagnosis was pneumonia (47.5% and 42.9%, respectively). There were no significant differences in children’s clinical presentations and diagnoses. Meanwhile, no family clustering and reinfection of CoVs were found in this study.

**Table 3:** Clinical characteristics of common HCoV infections: comparison between single detection and viral codetection

Parameter	Total HCoV infections (%, n = 124)	Single HCoV infections (%, n = 40)	HCoV coinfections (%, n = 84)	*P* value
Clinical symptoms-no. (%)				0.978
Cough	113 (91.1)	34 (85.0)	79 (94.0)	0.081
Fever	73 (58.9)	25 (62.5)	48 (57.1)	0.072
Expectoration	10 (8.1)	3 (7.5)	7 (8.3)	0.081
Wheeze	19 (15.3)	6 (15.0)	13 (15.5)	0.078
Haemoptysis	5 (4.0)	3 (7.5)	2 (2.4)	0.063
Rash	2 (1.6)	–	2 (2.4)	0.184
Admitting diagnosis-no. (%)				0.937
Pneumonia	55 (44.4)	19 (47.5)	36 (42.9)	0.072
Bronchopneumonia	48 (38.7)	15 (37.5)	33 (39.3)	0.079
Mycoplasma pneumonia	4 (3.2)	1 (2.5)	3 (3.6)	0.094
Bronchitis	5 (4.0)	1 (2.5)	4 (4.8)	0.109
Bronchiolitis	6 (4.8)	1 (2.5)	4 (4.8)	0.120
Viral pneumonia	2 (1.7)	–	2 (2.4)	0.184
Uncertainty	4 (3.2)	3 (7.5)	1 (1.2)	0.094

## Discussion

In this study, our findings aligned with research conducted in other regions whereby OC43 is the most frequently detected HCoV, with detection rate 36.3% (45/124) [14, 16, 18, 31–33]. The reason may be that OC43 induces an immune response to suppress infection by NL63, 229E or HKU1 [14, 26]. However, there are some results from some regions and countries inconsistent with us, with NL63 is the most common HCoV, such as Zhejiang province, Hong Kong, Japan, etc. [34–36]. As the previously observation of studies has been reported, the detection frequency of 229E is relatively lower comparing with other common HCoVs [15, 22].

Our data indicate that children < 2 years old are the main population of common HCoVs infection, which is consistent with the results of previous studies in other regions [4, 35]. In addition, we also found that the highest positive rates of these four HCoVs among children were all aged ≤ 6 months and 12–23 months, which suggests that the age distribution of children with HCoV infection is not associated with HCoV species. Our results also show that there is significantly more males than females among all HCoV-infected children, which is in agreement with previous findings [16, 18, 37]. These findings suggested that boys are more susceptible to HCoVs than girls. The observed sex variations could potentially be attributed to genetic differences. Type I interferons (IFN) are known to have a crucial role in the innate immune responses of the host against viral infections [38]. It is noteworthy that the X chromosome expresses TLR7, a prevalent sensor for viruses, resulting in women exhibiting more robust type I IFN responses when stimulated by TLR7 ligands [39].

The common HCoVs seasonality may results from the combination of viral, host, and environmental factors [40]. In general, the common HCoVs tend to be detected during the winter month in temperate-climate countries predominantly [3]. Our results indicate that common HCoVs were mainly prevalent in spring and autumn, which is consistent with other reports in China, but inconsistent with some other countries [14–16, 18, 25, 35, 41]. Therefore, studies which include more regions and cover longer time span are still necessary in China. Only then can we understanding the transmission of these four HCoVs better, and develop effective prevention and control strategies. Meanwhile, several studies pay attention to the seasonal patterns [42–44], and our findings also showed that OC43, NL63, and 229E displayed marked seasonality. Of these, OC43 caused diseases predominantly during spring and autumn, different with earlier studies which have shown it predominantly prevent in winter [16]. Consistent with the tropics [45], NL63 mainly detected during summer and autumn and rarely in spring and winter, but a study in Guangzhou have shown that NL63 infection mainly occurs in summer and winter [46]. 229E predominantly detected during spring and winter, which is partly consistent with the results of other studies [16, 46]. As for HKU1, the detection rate was high throughout the year except summer, which contrasts with some earlier findings [4, 16]. These results further show that the frequency of detection of the common HCoVs is difference in different regions or countries, and the seasonal epidemic characteristics of common HCoVs are greatly affected by temperature and geographical environments [40].

We investigated the prevalence pattern of these four HCoVs for 6 consecutive years, from 2014 to 2019. In concordance with previous studies [4, 14, 16, 18, 31, 47], we found that different HCoV types were predominant in different years: OC43 peaked annually from 2015 to 2019, while NL63, 229E and HKU1 had pronounced peaks in one or two years in 2015 or 2018. This suggested that individual species may only display peak activity every 2–3 years [4]. Furthermore, we also found that the detection rate of these four HCoVs was all higher in 2015 and 2018. We speculate that there were outbreaks in 2015 and 2018 in many Chinese areas. At the same time, the detection rate of individual HCoV infection may exhibit independent fluctuations, while the presence of cross-immunity within or between α-coronavirinae (NL63 and 229E) and β-coronavirinae (OC43 and HKU1) could potentially impact the detection rate of all four HCoV infections [15].

In our study, the detection rate of HCoV in Guangzhou was the highest, followed by Yinchuan, Wenzhou, Chongqing, and Beijing, while the other areas in our study (Guiyang, Shenyang, Hangzhou) were lower. The detection rate of HCoV infection in southern China was higher than that in northern China, which may be due to the number of hospitals was higher in southern China (5/8). Furthermore, OC43 infection was mainly concentrated in Beijing and Yinchuan, while HKU1 infection was mainly concentrated in Guangzhou and Wenzhou. Thus, OC43 might be more susceptible to epidemics in northern China, and HKU1 might be more susceptible to epidemics in southern China.

The common HCoVs were most commonly co-detected with other respiratory viruses [44]. In our study, of the 124 HCoV-positive patients, 40/124 (32.3%) were identified as involving only one virus, with NL63 was the most frequently mono-detected, while 229E was the most frequently co-detected. A pervious study in Shanghai also confirmed that 229E was the most frequency HCoV associated with codetection [14]. However, some earlier studies indicate that 229E was more frequently single-detected and HKU1 was more frequently co-detected] [48]. This may be due to a smaller number of cases of 229E. Moreover, a total of 84/124 (67.7%) HCoV-positive patients were codetected with one or more other respiratory viruses, and dual detection (64/124, 51.6%) were the most common detection patterns. Interestingly, only OC43 was codetected with other HCoVs, but no NL63-HCoV was detected. Similar to previous studies [15, 18], of the 9 codetected pathogens, the most commonly detected was EV/RhV, followed by HBoV, RSV and PIV. The most common codetected pathogen of OC43, NL63 and 229E was EV/RhV, while that of HKU1 was HBoV. Some reports indicated that coinfection probably influence the clinical characteristics of common HCoVs positive patients and associated with more severe diseases and higher mortality [14, 18, 44].

A number of studies showed, children with HCoV infection presented some pronounced respiratory symptoms, with cough (113/124, 91.1%) and fever (73/124, 58.9%) were the most common symptoms [18, 49]. Although once recognised as a “common cold” virus, an increasing number of studies have recognised HCoV as a respiratory virus associated with a wide range of upper respiratory tract infections and lower respiratory tract infections [2–6, 27, 50]. In our study, we verified findings from other studies that pneumonia and bronchopneumonia were the most common clinical diagnoses associated with common HCoVs [15, 16, 18]. Of these, OC43 and 229E were more associated with bronchopneumonia, while NL63 and HKU1 were more associated with pneumonia. In addition, there were no significant differences in the clinical presentations and clinical diagnoses between single HCoV detected and HCoV coinfection, which suggested that the severity of the disease was not associated with virus codetected, which is consistent with the study in Korean children [16]. However, as mentioned above, there were also reports that coinfection may influence the clinical presentations of HCoV-infected patients. Therefore, it remains unclear whether coinfection is associated with the severity of the disease.

However, although it included a long duration and large sample size, our study also has some limitations. First, we only admitted children who were hospitalised. Therefore, the results of our study may not necessarily reflect the overall burden of common HCoV infections in the paediatric population. Second, this study has unavoidable bias due to it being a retrospective study and the lack of healthy subjects. In addition, the hospitals studied may not be representative of those in the region, and the number of hospitals in our study is different between different regions.

## Conclusion

In this study, common HCoVs are one of the frequently detected respiratory pathogens of children in China. OC43 was the most commonly detected HCoV. We find that children are more susceptible to infection during spring, especially those who are under 2 years old. The detection rate of the four HCoVs was higher in southern China; however, OC43 and HKU1 were higher in northern and southern China, respectively. From our results, EV/RhV was the most commonly codetected pathogen. The most common symptoms were cough and fever, and the most common diagnoses were pneumonia and bronchopneumonia. Overall, common HCoVs are important in hospitalised children with respiratory pathogen infections in China. Our data provide further insight into the epidemiological, clinical and codetection features of four HCoV strains in paediatric patients.

## Data Availability

Not applicable.
